# Language assistance for limited English proficiency patients in a public ED: determining the unmet need

**DOI:** 10.1186/s12913-018-3823-1

**Published:** 2019-01-22

**Authors:** Breena R. Taira, Aristides Orue

**Affiliations:** Department of Emergency Medicine, North Annex, Olive View-UCLA Medical Center, 14445 Olive View Drive, Sylmar, CA 91342 USA

## Abstract

**Background:**

Many patients who present to public Emergency Departments (EDs) have Limited English Proficiency (LEP). LEP patients have worse understanding of their conditions and high rates of ED recidivism. LEP patients are entitled to language assistance under Title IV of the 1964 Civil Rights Act. The objective of this study is to characterize the unmet need for language assistance in a public ED.

**Methods:**

Retrospective chart review of 48 h of successive patient encounters in a public ED. Registration workers asked each patient their preferred language and whether they would like an interpreter. On recent implementation of a new electronic health record (EHR), however, providers were unable to see the responses recorded. When discovered, this created a natural experiment to compare patient request for language assistance with documented practice of the providers who were unaware of the patient’s stated preference at registration. The study was set in a public, urban ED, annual census of 50,000 visits, with language assistance services available 24/7 via video units and phone line. The subjects included all patients presenting to the ED for a 48-h period. Those with altered level of consciousness and those who left before being seen were excluded. Age, race, ethnicity, preferred language, preference for language assistance, status of the provider as certified bilingual, documentation of language assistance use, type of language assistance used (video, phone, bilingual staff or ad hoc) were captured. Descriptive statistics were used with proportions and 95% CIs to describe the unmet need.

**Results:**

In total, 253 encounters met inclusion criteria. Mean age was 41 years, 201/253 (79.5%) were Hispanic or Latino, and 134/253 (53%) preferred to use a language other than English (97% Spanish, 2% Armenian and 0.8% Tagalog). Of the 110/253 (43%) patients requesting an interpreter, 12/110 (10.9%) were seen by a certified bilingual provider and 5/110 (4.6%) had written documentation by the primary provider that language assistance was used. The calculated unmet need for spoken language assistance was 93/110 (84.5%) of patients requesting language assistance or 93/253 (36.8, 95%CI 31–42.9%) of total ED patients.

**Conclusions:**

In this public ED, there is a large unmet need for language assistance for LEP patients.

## Background

More than 60 million people in the United States speak a non-English language at home and of those, 42% self-report that they speak English less than “very well” and are considered to have limited English proficiency [1]. Although variability exists in the proportion of limited English Proficiency (LEP) patient encounters throughout the country, in many areas, LEP residents comprise the majority of the population [2], and thus the majority of patients seen in the Emergency Department.

It is well documented that LEP patients have worse outcomes than their English proficient counterparts. LEP patients have less understanding of their medical conditions [3] and higher rates of ED recidivism [4], likely secondary to poor understanding of their care. Under Title IV of the 1964 Civil Rights Act, patients are entitled to language assistance [5]. Multiple studies in the 1990’s and early 2000’s demonstrated the need for and importance of language assistance for LEP patients in the Emergency Department [3, 6–8]. Since then, however, underutilization of language assistance during LEP patient visits has been a persistent problem [9–12].

In a busy Emergency Department (ED), communication is of the utmost importance, but in setting of unscheduled care, assuring the right amount of language services available at the right time poses a challenge. The planning of interventions to improve language assistance in the ED requires both documentation and quantification of the current unmet need.

## Objectives

The objective of this study is to characterize the current unmet need for language assistance in a public ED by describing the proportion of those patients that request an interpreter that do not have documentation of language assistance use in their medical records. The secondary objective is to describe the unmet need for language-concordant patient education materials and discharge instructions.

## Methods

This study is a retrospective chart review of all patients presenting to a public Emergency Department during a 48-h period starting at midnight on July 1, 2016. The time-period was chosen because it was 6 months after the implementation of a new electronic health record (EHR), thus difficulties in navigating the EHR itself would not be expected to hinder documentation. In addition, the new interns for the academic year had not yet started clinical shifts and, therefore, all resident providers whose documentation would be reviewed had been working in this hospital for, at minimum, 1 year.

### Study design and setting

We performed a retrospective chart review of all patients registering for treatment in the ED during the designated 48-h time-period. The setting is a public ED with an annual census of 50,000 visits. The hospital is part of the Department of Health Services for the County of Los Angeles and a university affiliate with a 4-year residency training program in Emergency Medicine. It is the only public hospital for the San Fernando Valley in northern Los Angeles County. More than 50% of Los Angeles County speaks a non-English language at home, the most common languages being Spanish, Chinese, Tagalog, Korean, Armenian, Vietnamese, Farsi, Japanese and Russian [2]. The hospital subscribes to a language assistance service that provides spoken language assistance in 240 languages. There are 8 video-interpreter machines present in the 50 bed Emergency Department that are portable and can be wheeled into any patient room. In addition, an interpreter phone line can be accessed from any phone in the hospital. The hospital also has a system to certify bilingual employees. Employees who speak any of the target languages (those spoken by over 5% of the population) can take an exam in that language. If they receive a passing evaluation, they are then “certified” to perform their job in both languages. The exam is not role-specific, nor does it test any medical terminology. These employees are not given training in interpretation (assistance in spoken language) or translation (assistance in written language) and thus are not meant to act as in-person interpreters. The program is voluntary and incentivized and approximately 18% of ED staff are certified.

### Subjects

All patients presenting to the Emergency Department during the designated time-period were eligible to be included in the study. If the patient was unconscious, severely altered, or intubated immediately on arrival the patient was excluded as preferred language and request for an interpreter would not be relevant. If the patient completed the initial registration but left before being seen by a provider or left before their treatment was completed, they were excluded from the analysis as the documentation would not represent a full encounter. Because we planned to review discharge documentation, we also defined discharged patients as those who were discharged from the Emergency Department to home (i.e. not to the psychiatric emergency department or transferred to another hospital). This study was approved by the Olive View-UCLA Medical Center Institutional Review Board prior to the commencement of any research.

### Measures and outcomes

We generated a report from the electronic health record with the medical record numbers of all patients arriving to the emergency department for evaluation from 00:00 of July 1, 2016 for a 48- h period in addition to basic demographics. Other variables obtained in this report included age in years, race (White, Black, Asian, American Indian, Other or not reported), and ethnicity (Hispanic or Latino, Not Hispanic or Latino, and not reported), and primary language and discharge disposition (home, admit, Left Without Being Seen (LWBS), or Left Before Treatment Complete (LBTC)). The report also contained a variable for interpreter requested (yes/no) collected by registration workers after an initial Rapid Medical Exam by a healthcare provider. The exact question asked by the registration worker was “What is your preferred language?” and if the response was anything other than English, the follow up question was “Would you like an interpreter during your visit today?” During the time-period of this study, this information was documented in the registration view of the EHR, which is not accessible to the providers. The providers would thus would have to identify the need for language assistance themselves. Of note, this was a new data point that registration workers began asking with the implementation of the EHR, therefore, there was no history of providers relying on this field in the past, it was always the case that the providers were expected to identify LEP patients themselves. The rate of documented language assistance use was the primary outcome. The secondary outcome was the rate at which LEP patients received language concordant patient education materials and discharge instructions.

### Data abstraction

All charts were reviewed by the investigators using a pre-determined data abstraction form. Both investigators are fluent in Spanish and hold bilingual certification (Spanish/English) from the Department of Health Services of the County of Los Angeles and are qualified to review written documentation given to the patients in Spanish. Definitions of each variable were determined prior to data abstraction. Any conflict or uncertainty regarding whether the chart author was referring to language assistance use would be resolved in the favor of the provider (coded as yes for providing language assistance) as a mechanism to minimize investigator bias.

We reviewed all NP and MD notes for those patients who requested an interpreter in search of documentation of language assistance use. We defined language assistance in broad terms to capture all potential references. Any mention of interpreter, translator, help with language, language used etc. were recorded as use of language assistance. When documented, we also captured the type of assistance (video, phone, bilingual staff, ad hoc interpreter or unknown). Within the ED History and Physical template in our EHR, two fields of interest exist. The beginning of the template contains a pre-set list of potential communication barriers for which one option is “language barrier”. We abstracted whether language barrier was chosen by the author as an existing communication barrier (yes/no). In addition, the template contains choices for “history source” for which one option is “interpreter”. We recorded (yes/no) whether interpreter was chosen as the history source.

The name of the primary author of the history and physical was cross-checked against the county government’s list of “certified bilingual providers”. If the name was on the certified bilingual provider list and their certified language matched that of the patient, then the primary provider was considered bilingual during this encounter and would not be expected to access language assistance. The primary provider was defined as the principal author of the history and physical, be it a nurse practitioner, resident physician, or attending physician. If the attending physician was certified bilingual and co-signed a note that was primarily authored by another provider who was not certified bilingual, this was considered not bilingual, as presumably the primary provider (NP or resident) spent the most time with the patient and that interaction would have warranted language assistance.

The same was captured for nursing documentation. Nursing documentation of the triage and the initial assessment after the patient was placed in an ED room were reviewed as these are the two most extensive nursing notes and are required for all encounters.

For those patients discharged home from the ED, all discharge documentation was reviewed. Discharge instructions were divided into two categories. The first category of discharge instructions included pre-written diagnosis-specific education (for example, “upper respiratory infection”) chosen from a drop-down menu within the EHR. These will be referred to as “pre-printed education”. The second category is the patient-specific discharge instructions that must be written out by the provider (for example, “Go to the ophthalmology clinic tomorrow at 8am and bring your records”). These are referred to as “free-text instructions”. Both pre-printed education and free-text instructions were evaluated for language concordance. For both pre-printed education and free-text instructions, if the language of the text matched the patient’s preferred language, this was recorded as language concordant. For all free-text instructions in Spanish, the verbatim text written to the patient was recorded on the data abstraction form. These free-text instructions in Spanish were then reviewed for quality. We evaluated the instruction text and determined whether there were gross errors in the Spanish language used (yes/no). The definition of “gross errors” was pre-defined. Obvious mistranslation of terms, syntax errors and use of English language abbreviations in the Spanish text were all classified as “gross errors”. Missing accent marks or spelling errors were not considered “gross errors”.

### Analysis

Descriptive statistics were used for demographic characteristics of the charts reviewed. The unmet need for both spoken and written language assistance was described as the proportion of those with no documentation of language assistance use in their medical record to those requesting an interpreter and to the overall number of ED encounters. This was described as a proportion with 95% confidence intervals. Interrater reliability of the quality of Spanish language discharge instructions was assessed using the kappa statistic.

## Results

**Fig. 1:**
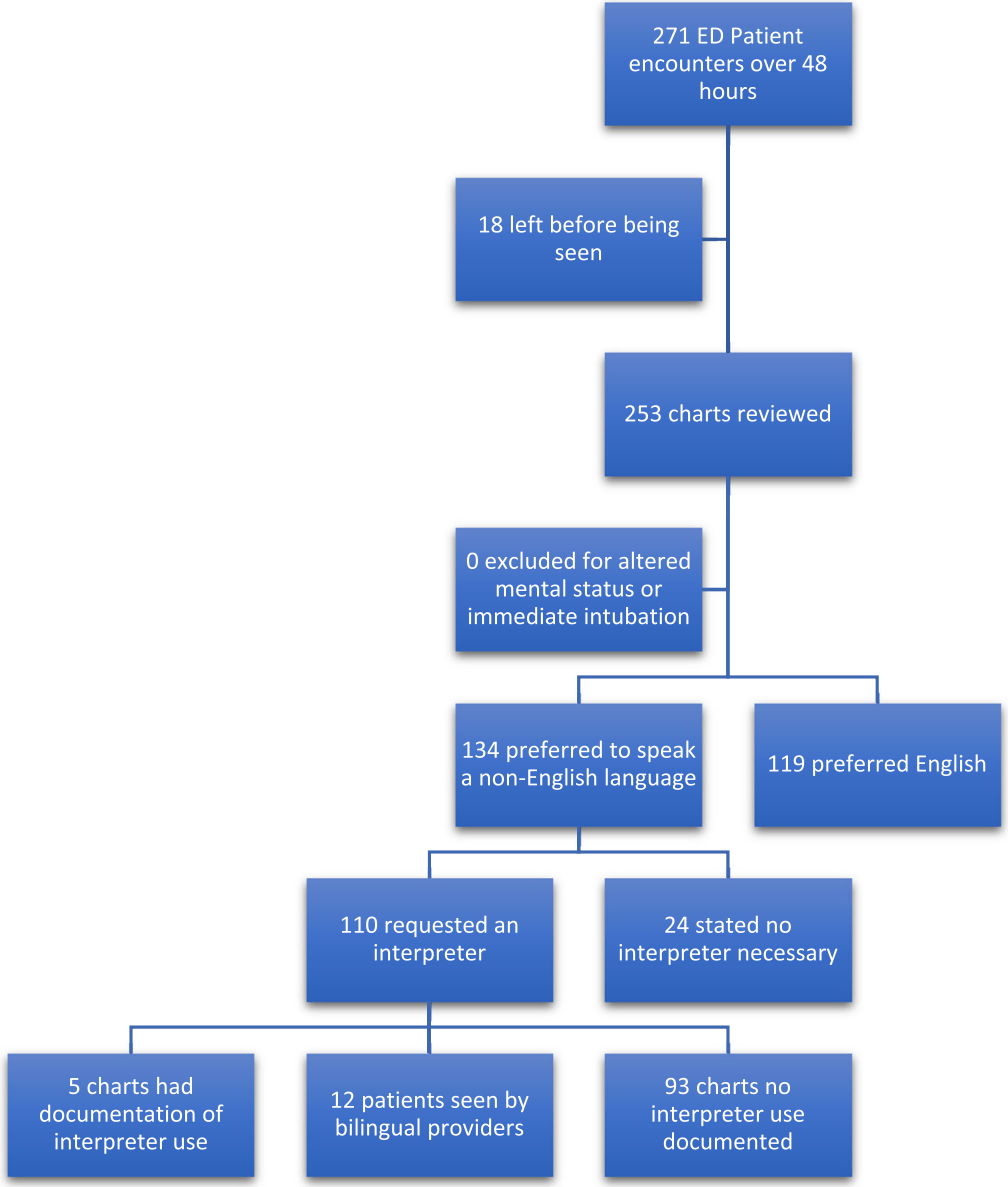
Patient flow diagram for spoken language needs

In total, 271 patients registered for care in our ED during the designated time-period. Of these, 18 left before being seen or before treatment was completed, leaving 253 patients with charts available for analysis. The mean age was 41 years and 201/253 (79.5%) were Hispanic or Latino. In total, 134/253 (53%) preferred a language other than English (97% Spanish, 2% Armenian and 0.8% Tagalog) and 110/253 (43%) requested an interpreter. (See Fig. 1) 12 of the patients (10.9%) who requested an interpreter were seen by a certified bilingual provider whose language matched the patient’s preferred language. Of the remaining patients who requested an interpreter, only 5/98 (5.1%) had medical records with documentation by the primary provider that language assistance was used. In all five charts, mention of the interpreter was found in the pre-populated template statement that listed “Interpreter” as the history source. None of the providers mentioned language assistance outside of this statement, thus there was also no information on the type of language assistance used and no one recorded the interpreter’s identification number given at the beginning of each video and phone encounter. In two charts, the choice “language barrier” was listed as a history limitation. Of those two, one chart also chose the interpreter as the history source, the other did not mention any means of overcoming the barrier. None of the nursing documentation reviewed mentioned language assistance use. The calculated unmet need for spoken language assistance in our ED was 93/110 (84.5%) of patients requesting language assistance or 93/253 (36.8, 95%CI 31–42.9%) of total ED patients.

**Fig. 2:**
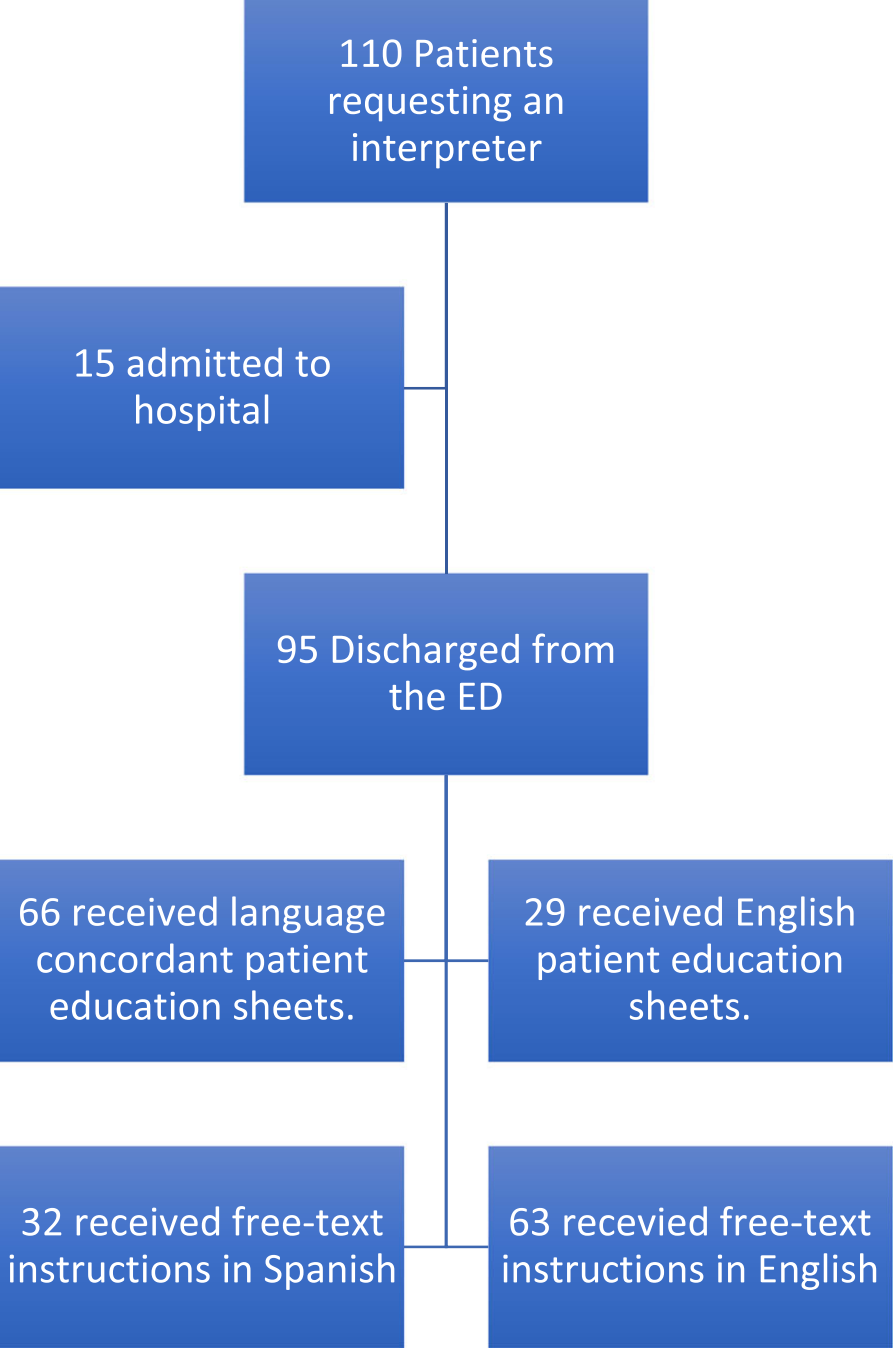
Flow diagram of disposition and written translation needs at discharge

Of the 110 patients requesting language assistance, 95 were discharged from the ED. (See Fig. 2) All discharge documentation for these 95 patients was reviewed. Of these, 66/95 (69%) received language concordant pre-written patient education sheets. In addition, 32/95 (33.7%) received free-text, patient-specific instructions in Spanish, of which 24/32 (75%) had obvious errors in translation (kappa = 0.81). The unmet need for language concordant written instructions of any type was 29/95 (30%) of those requesting language assistance or 29/253 (11.5, 95%CI 8.1–16%) of total patient encounters.

## Discussion

The unmet need for language assistance for LEP patients in this public ED is high. Our study is unique in that each patient was asked specifically both their preferred language and whether they would like an interpreter during the visit. Prior studies have asked retrospectively or used proxies such as native language or English proficiency [13, 14]. English proficiency and preferred language are not always equivalent, nor can knowledge of either definitely identify the patient’s preference for an interpreter [15].In this study, 24 of the patients who stated they preferred another language declined an interpreter when offered. This underscores the need to ask the patient directly whether they would like an interpreter.

The high unmet need is consistent with reports from emergency departments in other health systems. In 2008 Ginde et al. surveyed patients from four Boston EDs and found that after discharge, 18% of LEP patients stated their encounter was interpreted by a profession interpreter [14]. In 1996, a survey of patients at a different public hospital in Los Angeles County found that no interpreter was used for 46% of the patients for whom an interpreter was thought to be necessary by either the patient or the practitioner [3]. Despite the 20-year interval and improvement of available language services in our health system, we found that the gap has widened. It is, of course, possible that more communication occurred in the patient’s language than was documented. It is unlikely that the phone and video interpreters were used at high rates and not documented as we obtained records from the contracted language services and could identify only 3 calls from that time-period originating from the ED. It is possible, however, that providers were using their own non-English language skills to supplement language assistance. Supporting evidence includes the use of direct quotes in Spanish in the medical record. For example, one triage nurse documented the chief complaint as “Tengo sueño no más.” (I’m just sleepy) and a few charts used Spanish language terms to describe the physical symptoms of the patients (for example “hormigueos” for numbness and “piquetes” for a pricking or stinging sensation). These quotes imply that at least part of the conversation between the provider and the patient took place in Spanish despite lack of documentation and the lack of provider certification. Provider use of non-English language skills without certification is problematic as providers are frequently unable to recognize their own skill level and limitations [16]. Even when accounting for the possibility that some amount of language assistance use occurred but was not documented and some providers may speak Spanish but are not certified, this cannot account for the entirety of the unmet need. Most likely, ad hoc interpretation (interpretation by untrained families or friends) accounts for a large part of the discrepancy. While ad hoc interpreters may be convenient, they poses a risk to patient safety given their lack of training [17]. In addition, under section 1557 of the Affordable Care Act, LEP patients are entitled to a “qualified interpreter”, defined as someone who has proven abilities in English and the target language in addition to training in the ethics of interpretation [18]. Ad hoc interpreters do not meet this definition.

Beyond this, in some cases, we noted a specific avoidance of formal language assistance. In one case, a resident documented that, as he attempted to discharge a patient, the patient requested to talk to someone who spoke better Spanish. The resident then documented that he called his attending (who did not hold bilingual certification) and asked him to talk with the patient rather than call an interpreter. This is consistent with prior literature on decision making which shows that providers consistently fail to utilize language assistance when it is available [9, 12, 19, 20]. The perception appears to be that language assistance is a burden on the provider, despite good evidence that language assistance can have positive impact on the care of LEP patients. Studies demonstrate both patient and provider benefit, including improved understanding of diagnosis and treatment [3], improved ED throughput [21], decreased resource utilization [7], and decreased cost [8]. Beyond this, there is a legal precedent that requires that language assistance be offered to LEP patients [22]. Title VI of the 1964 U.S. Civil Rights Act ensures that federal money does not support providers who discriminate based on race, color or national origin, which is interpreted to include those with LEP [5]. All health care facilities that receive financial subsidy from the government must provide language assistance to LEP patients. Lack of provider knowledge and hospital enforcement, however, contribute to the persistent underutilization of language assistance [23].

We also saw that technology is deeply intertwined with both the type and quality of language assistance and produces both positive and negative effects. It was the new EHR that made possible this natural experiment because the providers could not see the request for language assistance documented by registration. We have since remedied the problem by making the information available as an interpreter icon on the main ED patient tracking board for those patients who request an interpreter. The pre-programmed history and physical template of the EHR appears to positively impact some providers as a reminder to document interpreter use. All 5 of the charts that mentioned language assistance used the “interpreter” choice from a dropdown menu. Technologic prompts may be useful in helping providers to record interpreter use in the history and physical. Standardized diagnosis-specific patient education materials are available in several languages in the EHR and this resource was utilized more consistently than any other type of language support.

Patient-specific written instructions, however, were problematic. Interestingly, this also seems to be related to technology. Most errors in the free-text patient instructions appear to be secondary to use of automated translation software. English abbreviations fed into automated translation software were a common problem. For instance, the providers would type “US” for “Ultrasound” (i.e. “Your US was normal”). The resulting translation found in the patient’s discharge instructions is “Estados Unidos” (United States- your United States was normal). Beyond mistranslated abbreviations, language translation software frequently changes syntax leading to unintelligible sentences. Translation software often separates the name of our hospital (Olive View Medical Center) into pieces and the resulting Spanish reads “View Medical Center” with the word “Olive” randomly inserted into a different clause of the sentence. A 2014 study of the use of Google Translate in medical communication found that it was only 57% accurate overall [24]. There is, however, in our hospital, no other source for on-the-spot written translation for patient specific instructions.

This study is important because it describes the glaring incongruity between patient preference and provider behavior regarding language assistance and that the gap is widening over time when compared to prior literature. The barrier that LEP patients face in accessing language assistance is us, the providers. We must rid ourselves of the idea that language assistance is a burden and embrace the evidence that it improves outcomes and is critical in providing high quality care for LEP patients. It is well established that health disparities exist within the United States along racial and ethnic lines [25]. Others have suggested that language barriers are a major contributor to these persistent disparities [26]. Our study supports this conclusion. When clinical practice patterns do not include the use of appropriate language assistance, as we report, the impact is not just to the individual but also to the entire population and is a feasible contributor to health disparities. As the U.S. becomes increasingly multilingual, not only does language assistance need to be available, but also education of providers needs to emphasize the ethical and legal imperative for the appropriate use of language assistance as part of a broader effort to achieve health equity.

### Limitations

A familiar adage tells us that “if it is not documented, it did not happen”. The assumption of any chart review is that the documentation is a reflection of the actual care provided [27]. Language assistance should presumably be recorded as this is the only way to confirm that the care provided meets the legal requirement to provide assistance. Documentation may not always reflect what is done, however, when documentation is uniformly absent, it is a warning sign that something is wrong on the system level. This review only sampled 48 h of patient encounters and the practices of the providers covering the ED during that time may not be generalizable to all providers. There is, however, no reason to believe that this sample is not representative and the findings reflect our experience. In addition, our findings may not be generalizable to all EDs. We have a very high rate of LEP patients compared to other institutions, however, overall numbers of LEP residents in the U.S. are on the rise and issues of language will become increasingly relevant to all U.S. healthcare institutions [1].

Although we assume that certified bilingual providers can communicate in their language of certification, this is not a given. As described in the methods section, the certification process is brief, not validated, and does not test any medical terminology. Other systems use more sophisticated certification processes [28, 29]. Partial fluency of providers is associated with worse patient understanding [30]. Further research and the establishment of a national standard for the safe use of non-English language skills in clinical medicine would contribute greatly to quality of care for LEP patients.

Finally, our bias as investigators, in this case, is also a limitation of the study. As two certified, bilingual (English/Spanish) emergency providers, we undertook the study with the idea that an underutilization of language assistance exists in our practice environment. We were careful to predefine all variables prior to data collection to minimize subjectivity.

## Conclusions

In conclusion, the unmet need for language assistance, both spoken and written, in this public Emergency Department is very high and signals an alarming disparity between patient preference for language assistance and provider behavior. Hospitals and Emergency Departments must consider not only spoken language assistance but also written language assistance for LEP patients. Technologic support and provider education are potential mechanisms to increase both use and documentation of language assistance. Addressing substandard communication with LEP patients is paramount because of its contribution to persistent health disparities on the population level. Further research is needed to identify which types of interventions (technologic, education, policy) are most effective at improving the appropriate use of language assistance as a step toward achieving health equity.

## Abbreviations

CI: Confidence Interval
ED: Emergency Department
LEP: Limited English Proficiency

## References

[1] Ryan C (2013). Language use in the United States: 2011. Bureau USC, ed.

[2] Kwoh S. LA Speaks: Language Diversity and English Proficiency by Los Angeles County Service Planning Area. https://advancingjusticela.org/sites/default/files/LASpeaksLanguageDiversity.pdf. Accessed 27 Dec 2018

[3] Baker DW, Parker RM, Williams MV, Coates WC, Pitkin K (1996). Use and effectiveness of interpreters in an emergency department. Jama.

[4] Ngai KM, Grudzen CR, Lee R, Tong VY, Richardson LD, Fernandez A (2016). The association between limited English proficiency and unplanned emergency department revisit within 72 hours. Ann Emerg Med.

[5] Title VI, Prohibition Against National Origin Discrimination Affecting Limited English Proficient Persons. National Archives and Records Administration, 2004. (Accessed 7 June 2017, at https://www.archives.gov/eeo/laws/title-vi.html.)

[6] Carrasquillo O, Orav EJ, Brennan TA, Burstin HR (1999). Impact of language barriers on patient satisfaction in an emergency department. J Gen Intern Med.

[7] Hampers LC, Cha S, Gutglass DJ, Binns HJ, Krug SE (1999). Language barriers and resource utilization in a pediatric emergency department. Pediatrics.

[8] Hampers LC, McNulty JE (2002). Professional interpreters and bilingual physicians in a pediatric emergency department: effect on resource utilization. Arch Pediatr Adolesc Med.

[9] Diamond LC, Schenker Y, Curry L, Bradley EH, Fernandez A (2009). Getting by: underuse of interpreters by resident physicians. J Gen Intern Med.

[10] Diamond LC, Tuot DS, Karliner LS (2012). The use of Spanish language skills by physicians and nurses: policy implications for teaching and testing. J Gen Intern Med.

[11] Lee JS, Napoles A, Mutha S, et al. Hospital discharge preparedness for patients with limited English proficiency: a mixed methods study of bedside interpreter-phones. Patient Educ Couns. 2018;101(1):25–32. 10.1016/j.pec.2017.07.026 PMC5732033 28774652

[12] Lee KC, Winickoff JP, Kim MK (2006). Resident physicians' use of professional and nonprofessional interpreters: a national survey. Jama.

[13] Karliner LS, Napoles-Springer AM, Schillinger D, Bibbins-Domingo K, Perez-Stable EJ (2008). Identification of limited English proficient patients in clinical care. J Gen Intern Med.

[14] Ginde AA, Sullivan AF, Corel B, Caceres JA, Camargo CA (2010). Reevaluation of the effect of mandatory interpreter legislation on use of professional interpreters for ED patients with language barriers. Patient Educ Couns.

[15] Gee GC, Walsemann KM, Takeuchi DT (2010). English proficiency and language preference: testing the equivalence of two measures. Am J Public Health.

[16] Schenker Y, Lo B, Ettinger KM, Fernandez A (2008). Navigating language barriers under difficult circumstances. Ann Intern Med.

[17] Hsieh E (2006). Understanding medical interpreters: reconceptualizing bilingual health communication. Health Commun.

[18] Federal Register/Vol. 81, No.96/Wednesday, May 18, 2016 /Rules and Regulations. This is a government report available at: https://www.govinfo.gov/content/pkg/FR-2016-05-18/pdf/2016-11458.pdf.

[19] Burbano O'Leary SC, Federico S, Hampers LC (2003). The truth about language barriers: one residency program's experience. Pediatrics.

[20] Ramirez D, Engel KG, Tang TS (2008). Language interpreter utilization in the emergency department setting: a clinical review. J Health Care Poor Underserved.

[21] Grover A, Deakyne S, Bajaj L, Roosevelt GE (2012). Comparison of throughput times for limited English proficiency patient visits in the emergency department between different interpreter modalities. J Immigr Minor Health.

[22] Chen AH, Youdelman MK, Brooks J (2007). The legal framework for language access in healthcare settings: Title VI and beyond. J Gen Intern Med.

[23] Youdelman MK (2008). The medical tongue: U.S. laws and policies on language access. Health Aff (Millwood).

[24] Patil S, Davies P (2014). Use of Google translate in medical communication: evaluation of accuracy. Bmj.

[25] Smedley BD, Stith AY, Nelson AR (2003). Unequal Treatment: Confronting racial and ethnic disparities in health care.

[26] Saha S, Fernandez A, Perez-Stable E (2007). Reducing language barriers and racial/ethnic disparities in health care: an investment in our future. J Gen Intern Med.

[27] Kaji AH, Schriger D, Green S (2014). Looking through the retrospectoscope: reducing bias in emergency medicine chart review studies. Ann Emerg Med.

[28] Moreno MR, Otero-Sabogal R, Newman J (2007). Assessing dual-role staff-interpreter linguistic competency in an integrated healthcare system. J Gen Intern Med.

[29] de Jaimes FN, Batts F, Noguera C, Guerrero L, Moreno G (2013). Implementation of language assessments for staff interpreters in community health centers. J Health Care Poor Underserved.

[30] Fernandez A, Schillinger D, Grumbach K (2004). Physician language ability and cultural competence. An exploratory study of communication with Spanish-speaking patients. J Gen Intern Med.

